# Can participation in sports enhance adolescents’ subjective wellbeing? The chain mediation role of social support and basic psychological needs

**DOI:** 10.3389/fpsyg.2026.1878247

**Published:** 2026-08-04

**Authors:** Lumin Liu, Dan Li, Huairan Wang, Hwang Jin

**Affiliations:** 1 Physical Education, Jeonbuk National University, Jeonju, Republic of Korea; 2 Physical Education College, Jilin University, Changchun, China

**Keywords:** adolescents, basic psychological needs satisfaction, social support, sport participation, subjective wellbeing

## Abstract

**Background:**

Adolescent mental health has become a major global concern. Subjective wellbeing, as a core indicator of mental health, requires exploration of its intervention pathways. Participation in sports, encompassing physical regulation, emotional release, and social interaction, is considered an important avenue to promote subjective wellbeing; however, its specific mechanisms remain unclear.

**Objective:**

This study aims to explore the relationships among sports participation, social support, basic psychological needs, and subjective wellbeing, providing a theoretical basis for enhancing adolescents’ subjective wellbeing.

**Methods:**

A survey was conducted among 1,000 adolescents from Shandong, Henan, Jilin, Guangdong, Shaanxi, and Liaoning using scales measuring sports participation, social support, basic psychological needs, and subjective wellbeing.

**Results:**

Sports participation significantly and positively predicted both social support and basic psychological needs. Social support and basic psychological needs served as partial mediators in the relationship between sports participation and adolescents’ subjective wellbeing, with a significant chain mediation effect observed.

**Conclusion:**

The chain mediation pathway of social support and basic psychological needs indicates that sports participation enhances adolescents’ subjective wellbeing by creating a supportive social environment, which satisfies basic psychological needs. Furthermore, moderate levels of physical activity were associated with the subjective wellbeing, suggesting that only appropriately dosed exercise effectively promotes individual wellbeing, while insufficient or excessive exercise may diminish the impact of sports participation on subjective wellbeing.

## 1 Introduction

Adolescent mental health has become one of the core challenges in global public health. According to World Health Organization [WHO] (2025), a substantial number of adolescents worldwide are experiencing varying degrees of mental health difficulties, most of whom do not receive timely interventions. With economic development and increasing educational pressures, adolescents face multiple stressors related to academics, interpersonal relationships, and more, which are closely linked to declines in subjective wellbeing (Schmidt and Hansson, 2024). The National Health Commission of the People’s Republic of China (2018) reported that approximately 30 million adolescents in China suffer from emotional disorders. No, despite improvements in material conditions, the prevalence of “psychological distress” among adolescents has become increasingly prominent, indicating that material satisfaction alone is insufficient to effectively promote mental health. More seriously, if adolescent mental health issues are not addressed in time, they are likely to persist into adulthood, increasing the risk of various psychiatric disorders (Supke et al., 2021).

From the perspective of positive psychology, exploring pathways to enhance adolescents’ subjective wellbeing is of significant importance. Sports participation, as an economical and efficient approach, profoundly impacts individual subjective wellbeing (Guo et al., 2024). Psychologically, successful experiences during sports participation help enhance positive emotions, and the immersion experienced in physical activity is a key source of subjective wellbeing (Lubans et al., 2016). Physiologically, appropriate sports participation can promote the release of neurotransmitters such as dopamine, alleviating stress and improving mood (Ando et al., 2024). Socially, team sports provide adolescents with opportunities for social interaction, helping to establish positive peer relationships, gain social support, and enhance a sense of belonging and cooperation (Zhou et al., 2023).

Despite the well-documented positive association between sports participation and subjective wellbeing, several gaps remain in the existing literature. First, prior studies have largely focused on the independent effects of individual factors on subjective wellbeing, providing limited insight into the integrated pathways through which external environmental resources and internal psychological processes jointly contribute to wellbeing enhancement. Second, analyses based solely on average effects in the overall sample may overlook important boundary conditions that shape the relationship between sports participation and wellbeing. To address these limitations, the present study examines potential variations by gender, sport type, only-child status, and physical activity level. Finally, this study found that the relationship between physical activity volume and subjective wellbeing was not simply linear but instead exhibited an inverted U-shaped pattern. This finding challenges the traditional assumption that “more exercise is always better” and suggests that there may be an optimal range of physical activity participation for maximizing subjective wellbeing. Moreover, existing Self-Determination Theory (SDT) research has primarily focused on how social support and the satisfaction of basic psychological needs facilitate positive psychological outcomes, while relatively limited attention has been paid to the influence of behavioral intensity or dose on individuals’ psychological experiences and outcomes.

Against this background, the present study aims to provide a comprehensively examine the relationship between sports participation and adolescents’ subjective wellbeing. Specifically, it investigates the serial mediating roles of social support and basic psychological needs while also exploring whether the underlying mechanisms differ across demographic groups and sport-related contexts. By doing so, the study seeks to advance understanding of the psychological processes linking sports participation to wellbeing and to provide evidence for the development of sport-based wellbeing interventions among adolescents.

## 2 Research hypotheses

### 2.1 The association between sports participation and adolescents’ subjective wellbeing

Sports participation is widely recognized as a key factor in promoting subjective wellbeing (Buecker et al., 2021), with regular physical activity being associated with higher life satisfaction and positive affect. From a physiological perspective, physical exercise can stimulate the release of various neurotransmitters in the brain, including dopamine and endorphins, which are closely linked to pleasure and wellbeing (Heyman et al., 2012). In particular, the release of endorphins is widely considered to enhance adolescents’ positive emotions. Additionally, sports activities can improve adolescents’ sleep quality and reduce sleep disturbances, with high-quality sleep serving as a critical foundation for emotional health and life satisfaction (Yan et al., 2025).

From a psychological perspective, sports participation provides adolescents with abundant practical experiences, which can strengthen their sense of self-efficacy. According to Bandura and Wessels (1997) self-efficacy theory, the belief in one’s abilities formed through successful experiences enhances one’s sense of control over life, thereby promoting subjective wellbeing. Furthermore, sports participation effectively facilitates adolescents’ emotional regulation abilities. A review by Biddle and Asare (2011) indicated that regular physical exercise can significantly reduce the incidence of depression and anxiety among adolescents while increasing experiences of positive emotions.

Existing research has demonstrated that sports participation can be positively associated with individuals’ subjective wellbeing (Wicker and Frick, 2017). Based on this, the present study proposes:

*H1*: Sports participation is positively related to adolescents’ subjective wellbeing.

### 2.2 The mediating role of social support between sports participation and adolescents’ subjective wellbeing

Sports participation, particularly team sports, is considered an important source of social support for adolescents (He et al., 2026). The inherent interactive and collaborative nature of sports provides adolescents with abundant social opportunities. During the process of completing training tasks and devising competition strategies, individuals need to communicate and cooperate with teammates and coaches. This high-frequency interaction helps adolescents expand their social networks and establish new social connections (Li, 2025). Compared with general social contexts, sports participation is often accompanied by a higher level of emotional investment, as individuals strive and work hard to achieve common goals. These shared efforts can significantly cultivate strong team cohesion and a sense of collective belonging (Kwon, 2024). When adolescents perceive themselves as indispensable members of a group and feel accepted, trusted, and valued by team members, their perceived level of social support increases significantly (Mtshweni, 2024). In addition, a review study has demonstrated a positive association between sports participation and adolescents’ social support (Eime et al., 2013).

Social support serves as a critical buffer against life stressors and negative events (Cohen and Wills, 1985). During adolescence, individuals inevitably face challenges such as academic pressure, interpersonal conflicts, and competitive failures, which may trigger negative emotions such as depression and frustration (Rueger et al., 2016). According to the stress-buffering model, when individuals perceive understanding, care, and assistance from others, they are more likely to regard stressful events as controllable, thereby mitigating the negative impact on mental health (Rodríguez-Rivas et al., 2023). Furthermore, social support can enhance adolescents’ positive psychological states. High levels of social support strengthen adolescents’ sense of belonging and acceptance, fostering positive emotional experiences during social interactions (Fabris et al., 2024). Being valued and cared for itself constitutes a profound experience of happiness, forming an important foundation for subjective wellbeing (Wu and Lee, 2022).

Based on this, the study proposes:

*H2*: Social support mediate the association between sports participation and adolescents’ subjective wellbeing.

### 2.3 The mediating role of basic psychological needs between sports participation and adolescents’ subjective wellbeing

According to self-determination theory (SDT), individuals possess three basic psychological needs: autonomy, competence, and relatedness. The satisfaction of these needs is not only crucial for individual behavior but also affects psychological health development (Vansteenkiste et al., 2020; Liu et al., 2025). Sports participation, as a form of systematic physical activity and social interaction, provides diverse opportunities to satisfy adolescents’ basic psychological needs (Van Yperen, 2025).

First, during sports participation, adolescents can experience a sense of decision-making and control over their actions by autonomously choosing sports, training schedules, and competition goals (Chen et al., 2025). This sense of autonomy enables adolescents to recognize that their behaviors are self-determined rather than externally controlled, thereby motivating continued engagement in physical activity. Second, through overcoming challenges, completing tasks, and making progress, adolescents can tangibly perceive improvements in their abilities (O’Neil and Smith, 2026). Finally, sports include both team and individual events, with team activities offering rich social opportunities (Wade et al., 2026). By overcoming difficulties together during competitions and supporting one another during training, adolescents can establish close relationships with peers. This sense of being valued by the group satisfies the intrinsic desire to form positive connections with others, enhancing the need for relatedness. McDonough and Crocker (2007) found that sports participation can satisfy adolescents’ basic psychological needs.

When the three basic psychological needs—autonomy, competence, and relatedness—are satisfied, individuals are more likely to experience positive emotions and higher life satisfaction, which are core components of subjective wellbeing (Drapeau et al., 2025). Specifically, the satisfaction of these needs promotes intrinsically motivated behaviors, allowing individuals to continually experience pleasure during activities (Vallerand and Losier, 1999). Additionally, meeting basic psychological needs enhances emotional regulation abilities, helping individuals maintain relatively stable and positive psychological states when facing stress (Cohen and Wills, 1985). Thus, the fulfillment of basic psychological needs can transform external environmental stimuli into internal positive emotions. When adolescents’ basic psychological needs are nurtured in sports contexts, high levels of intrinsic motivation are elicited, leading to increased life satisfaction and reduced negative emotions (Rueger et al., 2016).

In summary, the study proposes:

*H3*: Basic psychological needs mediate the association between sports participation and adolescents’ subjective wellbeing.

### 2.4 Chain mediation

The positive association between sports participation and subjective wellbeing has been confirmed by numerous scholars, yet its underlying mechanisms remain to be further elucidated. In this study, two mediating pathways are proposed: social support and basic psychological needs. Moreover, social support is also related to basic psychological needs. Specifically, social support is most directly associated with the satisfaction of the need for relatedness (Howard et al., 2023). Humans are social animals, and the desire for belonging is deeply rooted in our genes (Counted et al., 2025). When individuals feel cared for, accepted, and valued, their sense of belonging is greatly satisfied.

Furthermore, SDT posits that a supportive social environment can fulfill individuals’ basic psychological needs, stimulate the development of intrinsic motivation, and positively impact psychological health (Huang et al., 2024).

**FIGURE 1:**
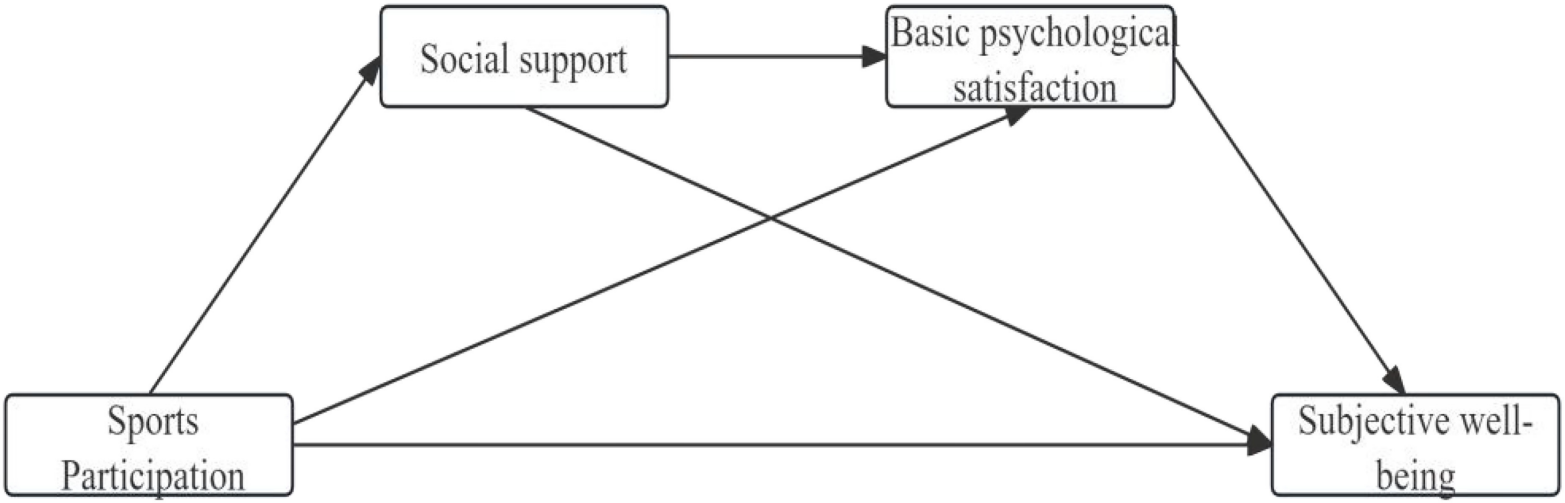
The conceptual model proposed in this study.

Integrating Hypotheses 1–3, it is proposed that there may exist a chain mediation pathway: “Sports Participation → Social Support → Basic Psychological Needs → Subjective Wellbeing.” The underlying mechanism by which sports participation predicts subjective wellbeing may be as follows:

Sports activities provide adolescents with abundant opportunities for social interaction, allowing them to establish social support networks. The social support gained through sports participation further nourishes individuals’ basic psychological needs. Ultimately, the fulfillment of these basic psychological needs directly promotes the generation of positive emotions, thereby significantly enhancing adolescents’ subjective wellbeing. Based on this, the study proposes:

*H4*: Social support and basic psychological needs serially mediate the association between sports participation and adolescents’ subjective wellbeing (Figure 1).

## 3 Research methods

### 3.1 Research participants

This study employs a cross-sectional questionnaire survey design and uses a non-probability convenience sampling, primarily because surveys targeting minors require informed consent from both schools and parents, making it difficult to implement strict probability sampling under practical conditions. To increase the heterogeneity of the sample, adolescents from 12 schools in the provinces of Shandong, Henan, Jilin, Guangdong, Shaanxi, and Liaoning were recruited as participants. A total of 1,100 questionnaires were distributed, with 1,090 returned, yielding a recovery rate of 99.09%. Among these, 1,000 were valid, corresponding to a validity rate of 90.91%.

Inclusion criteria for the sample were: (1) aged 10–19 years; (2) participation in sports activities within the past month; and (3) no severe physical or mental illnesses. The final valid sample consisted of 1,000 adolescents (504 males and 496 females).

The study was conducted through the schools’ physical education teaching and research groups. Questionnaires were distributed at the class level after parents provided informed consent, and participants completed the questionnaires anonymously. All procedures adhered to the ethical guidelines of the Declaration of Helsinki, and the study protocol was approved by the Ethics Committee of Jeonbuk National University (KIRD-2024-03-31-J-E-346).

### 3.2 Research instruments

#### 3.2.1 Physical Activity Rating Scale (PARS-3)

This study used the Chinese version of the Physical Activity Rating Scale (PARS-3), revised by Liang, 1994 to assess the level of sports participation. The scale is a widely used tool for measuring exercise behavior both domestically and internationally, demonstrating good reliability and validity.

The scale consists of three dimensions: intensity, frequency, and duration, rated on a 1–5 scale (1 = light activity, 5 = high-intensity sustained activity). Exercise intensity reflects the physiological load during exercise, exercise frequency refers to the number of exercise sessions per week, and duration indicates the length of each session. The overall physical activity level reflects the cumulative level of exercise participation over the past month. The calculation formula is: Physical Activity Volume = Intensity Score × (Duration Score-1) × Weekly Frequency Score. Higher values indicate greater participation in sports. Scores range from 0 to 100, with ≤19 indicating low activity, 20–42 indicating moderate activity, and ≥43 indicating high activity. In this study, the Cronbach’s α of the scale was 0.710.

#### 3.2.2 Social support scale

The study employed the Social Support Scale for Physical Exercise developed by Zhong et al., 2019. The scale includes 24 items divided into four dimensions: emotional support, informational support, instrumental support, and peer support, rated on a 5-point Likert scale. Higher overall or dimensional scores indicate higher levels of social support received in the context of physical exercise. In this study, the Cronbach’s α coefficients for the four dimensions were 0.842, 0.839, 0.848, and 0.855, respectively. Confirmatory factor analysis indicated good model fit (χ^2^/df = 1.521, GFI = 0.970, IFI = 0.990, TLI = 0.989, RMSEA = 0.023, PNFI = 0.867, PCFI = 0.883).

#### 3.2.3 Basic Psychological Needs Scale

The study used the Basic Psychological Needs Scale developed by Tao, 2024, which includes 12 items. Autonomy needs consist of 4 items, competence needs consist of 4 items, and relatedness needs consist of 4 items. In this study, the Cronbach’s α coefficients for the three dimensions were 0.815, 0.797, and 0.799, respectively. Confirmatory factor analysis supported a three-factor structure (χ^2^/df = 0.912, GFI = 0.992, IFI = 0.990, TLI = 0.999, RMSEA = 0.001, PNFI = 0.766, PCFI = 0.773).

#### 3.2.4 Subjective Wellbeing Scale (SWBS)

The study employed the Subjective Wellbeing Scale developed by Diener et al. (1985), which includes two parts: life satisfaction and affective emotions, using a 7-point Likert scale. Scoring method: The life satisfaction score is calculated by dividing the total score of items 1–5 by the number of items (5). The affective emotions section comprises items 6–19 and can be subdivided into positive affect and negative affect. The positive affect score (items 6–13) is calculated by dividing the total score of these items by 6; the negative affect score (items 14–19) consists of reverse-scored items, with the score obtained by dividing the total score of these items by 8. In this study, the Cronbach’s α coefficients for the three dimensions were 0.828, 0.886, and 0.862, respectively. Confirmatory factor analysis indicated good model fit (χ^2^/df = 1.590, GFI = 0.976, IFI = 0.991, TLI = 0.990, RMSEA = 0.024, PNFI = 0.852, PCFI = 0.864).

### 3.3 Research procedure

A total of 1,000 adolescents aged 10–19 from Shandong, Henan, Jilin, Guangdong, Shaanxi, and Liaoning were surveyed using questionnaires. After obtaining consent from school administrators, the research objectives were explained to the participants. Convenience sampling was used to administer the questionnaires to students. After collection, the data were organized in Excel and then entered into SPSS 26 and AMOS 26 for data processing.

### 3.4 Data analysis

Statistical analyses were conducted using SPSS and AMOS. Descriptive statistics (means and standard deviations) and Pearson correlation coefficients were first computed for all study variables. Independent-samples *t*-tests were subsequently performed to examine differences in physical activity participation, social support, basic psychological needs, and subjective wellbeing across demographic groups (e.g., gender, sport type, and only-child status). To test the hypothesized relationships among the study variables, structural equation modeling (SEM) was conducted. The proposed serial mediation model was further examined using the PROCESS macro with a bias-corrected bootstrap procedure based on 5,000 resamples. Indirect effects and their 95% confidence intervals (CIs) were estimated while controlling for demographic covariates. A mediation effect was considered statistically significant when the corresponding 95% CI excluded zero. All statistical tests were evaluated at the .05 significance level.

## 4 Research results and analysis

### 4.1 Common method bias test

This study employed Harman’s single-factor test to examine common method bias. All variables were included in a factor analysis. The results showed that there were 11 factors with eigenvalues greater than 1, and the largest factor explained 15.112% of the variance, which is below the standard threshold of 40% (Tang and Zhonglin, 2020). Furthermore, to provide a more rigorous examination of common method bias, four competing measurement models were constructed. As shown in Table 1, the hypothesized four-factor model demonstrated the best model fit (χ^2^/df = 1.458, CFI = 0.990, TLI = 0.987, RMSEA = 0.021, SRMR = 0.026), with all fit indices meeting the recommended criteria. In contrast, the three-factor, two-factor, and single-factor models exhibited substantially poorer model fit. Further comparisons revealed a progressive decline in model fit as the number of factors decreased, indicating satisfactory discriminant validity among the four constructs and suggesting that common method bias was unlikely to be a serious concern in the present study.

**TABLE 1:** The model fit index tests.

Items	χ^2^/df	CFI	TLI	RMSEA	SRMR
Four-factor model	1.458	0.990	0.987	0.021	0.026
Three-factor model	11.973	0.755	0.691	0.105	0.110
Two-factor model	28.130	0.374	0.237	0.165	0.173
Single-factor model	32.667	0.258	0.109	0.178	0.186

### 4.2 Demographic analysis of the sample

Descriptive statistical analysis was conducted on the sample, with results shown in Table 2. In this survey, there were 504 male participants and 496 female participants. The gender ratio was approximately 50% each, which somewhat reduces the potential impact of gender imbalance on the data results. Regarding educational level, adolescents were categorized as elementary school, junior high school, senior high school, and university. Elementary school students accounted for the largest proportion of 26.8%, followed by senior high school students at 25.8%, while junior high school and university students were the least represented, at 23.2 and 24.2%, respectively. In terms of place of origin, rural, suburban, and urban participants accounted for 34.4, 33.9, and 31.7%, respectively. The relatively higher proportion of rural participants reflects the strong national emphasis on rural development and the concept of rural revitalization, where education is increasingly seen as a means to change one’s destiny, and the accessibility and equity of education have been further improved. Regarding project categories, 504 participants (50.4%) participated in individual events, while 496 participants (49.6%) participated in team events. Concerning the status of being an only child, 495 participants (48.5%) were only children, and 505 participants (50.5%) were non-only children.

**TABLE 2:** Summary of basic information of participants (*n* = 1,000).

Project	Category	Count	Percentage
Gender	Male	504	50.4%
	Female	496	49.6%
Education level	Primary school	268	26.8%
	Junior high school	232	23.2%
	Senior high school	258	25.8%
	University	242	24.2%
Hometown	Rural area	344	34.4%
	Urban-rural fringe	339	33.9%
	Urban area	317	31.7%
Project type	Individual project	504	50.4%
	Team project	496	49.6%
Only child	Yes	495	48.5%
	No	505	50.5%

### 4.3 Correlation analysis among variables

This study conducted Pearson correlation analyses on core variables and their sub-dimensions to explore the relationships among the variables. As shown in Table 3, sports participation was significantly and positively correlated with subjective wellbeing (*r* = 0.091, *p* < 0.01), as well as with its sub-dimensions of positive affect (*r* = 0.072, *p* < 0.05) and life satisfaction (*r* = 0.080, *p* < 0.05), but was not significantly correlated with negative affect. Basic psychological need satisfaction was significantly and positively correlated with subjective wellbeing (*r* = 0.156, *p* < 0.001). Social support was significantly and positively correlated with both basic psychological needs (*r* = 0.075, *p* < 0.05) and subjective wellbeing (*r* = 0.111, *p* < 0.001). Overall, the correlation matrix provides preliminary support for the research hypotheses, showing expected positive associations among the main variables, which lays the foundation for subsequent path analysis.

**TABLE 3:** Correlation analysis among variables.

Variables	M	SD	1	2	3	4	5	6	7	8	9	10	11	12	13	14
Sports participation (1)	18.967	21.255	1	1	1	1	1	1	1	1	1	1	1	1	1	1
Subjective wellbeing (2)	3.011	1.097	0.091**													
Negative affect (3)	2.95	1.51	0.055	0.736***												
Positive affect (4)	2.896	1.453	0.072*	0.822***	0.338***											
Life satisfaction (5)	3.27	1.368	0.080*	0.676***	0.344***	0.356***										
Basic psychological needs (6)	3.026	1.204	0.089**	0.156***	0.092**	0.102**	0.182***									
Relatedness (7)	2.806	1.602	0.092**	0.230***	0.174***	0.151***	0.214***	0.705***								
Competence (8)	2.846	1.627	0.091**	0.217***	0.125***	0.142***	0.253***	0.728***	0.373***							
Autonomy (9)	3.104	1.553	0.082**	0.281***	0.172***	0.203***	0.283***	0.733***	0.453***	0.496***						
Social support (10)	2.202	0.796	0.099**	0.111***	0.094**	0.054	0.121***	0.075*	0.02	0.055*	0.062					
Companion support (11)	2.128	1.058	0.098**	0.033	0.013	0.007	0.072*	0.057	-0.005	0.034*	0.036	0.749***				
Instrumental support (12)	2.15	1.056	0.067*	0.049	0.051	-0.007	0.093**	0.074*	0.052	0.061	0.054	0.751***	0.384***			
Informational support (13)	2.149	1.041	0.073*	0.098**	0.095**	0.052	0.084**	0.062	0.02	0.043	0.063*	0.761***	0.405***	0.407***		
Emotional support (14)	2.38	0.977	0.068*	0.167***	0.135***	0.120***	0.127***	0.037	-0.007	0.031*	0.037	0.823***	0.511***	0.517***	0.535***	

*p < 0.05,

**p < 0.01,

***p < 0.001.

### 4.4 Analysis of differences in gender, sport, whether the individual is an only child, and level of physical activity

#### 4.4.1 Analysis of gender differences

Table 4 presents the results of the independent-samples *t*-tests by gender. No significant gender differences were found in sports participation, social support, basic psychological needs, or subjective wellbeing (all *p* > 0.05). These findings suggest that male and female adolescents reported similar levels across all core study variables.

**TABLE 4:** Analysis of gender differences.

	Gender (M ± SD)		*t*	*p*
Variables	Male (*n* = 504)	Female (*n* = 496)		
Sports participation	18.79 ± 20.31	19.15 ± 22.19	–0.272	0.786
Social support	2.17 ± 0.79	2.23 ± 0.80	–1.204	0.229
Basic psychological needs	3.06 ± 1.20	2.99 ± 1.21	0.984	0.325
Subjective wellbeing	3.01 ± 1.10	3.01 ± 1.09	0.003	0.997

*p < 0.05, **p < 0.01, ***p < 0.001.

#### 4.4.2 Analysis of differences between sport type

Table 5 presents the results of the independent-samples t-tests comparing adolescents participating in individual and team sports. No significant group differences were found in sports participation, social support, basic psychological needs, or subjective wellbeing (all *p* > 0.05). These findings suggest that adolescents involved in individual and team sports reported comparable levels across all core study variables.

**TABLE 5:** Analysis of differences between sport type.

	Sport type (M ± SD)		*t*	*P*
Variables	Individual events (*n* = 504)	Team events (*n* = 496)		
Sports participation	18.87 ± 21.08	19.06 ± 21.46	-0.141	0.888
Social support	2.22 ± 0.80	2.18 ± 0.79	0.727	0.467
Basic psychological needs	3.07 ± 1.21	2.98 ± 1.19	1.159	0.247
Subjective wellbeing	3.00 ± 1.08	3.02 ± 1.12	-0.315	0.753

*p < 0.05, **p < 0.01, ***p < 0.001.

#### 4.4.3 Group differences by only-child status

Table 6 presents the results of the independent-samples t-tests by only-child status. A significant group difference was observed for social support, with only children reporting higher levels of social support than non-only children (*t* = 2.118, *p* = 0.034). No significant differences were found for sports participation, basic psychological needs, or subjective wellbeing (all *p* > 0.05).

**TABLE 6:** Group differences by only-child status.

	Only-child status (M ± SD)		*t*	*P*
Variables	Yes (*n* = 495)	No (*n* = 505)		
Sports participation	19.85 ± 22.57	18.10 ± 19.87	1.303	0.193
Social support	2.26 ± 0.79	2.15 ± 0.80	2.118	0.034*
Basic psychological needs	2.99 ± 1.21	3.06 ± 1.20	-0.978	0.328
Subjective wellbeing	2.99 ± 1.08	3.03 ± 1.11	-0.482	0.630

*p < 0.05, **p < 0.01, ***p < 0.001.

#### 4.4.4 Analysis of differences in subjective wellbeing by level of sports participation volume

To examine differences in subjective wellbeing across exercise-volume groups, a one-way ANOVA was conducted to compare the low, moderate, and high exercise volume groups, followed by post hoc multiple comparisons using the LSD method (Table 7). The results showed that the moderate exercise volume group had the highest score for subjective wellbeing (M ± SD = 3.23 ± 0.95), followed by the high exercise volume group (M ± SD = 3.00 ± 1.05), and the low exercise volume group had the lowest score (M ± SD = 2.89 ± 1.15). The LSD post hoc test further revealed that all pairwise comparisons among the three groups were statistically significant (all *p* < 0.001): the moderate exercise volume group scored significantly higher than both the high and low exercise volume groups; the high exercise volume group also scored significantly higher than the low exercise volume group. These findings indicate a differential association between the level of sports participation and subjective wellbeing, with the moderate exercise volume corresponding to the highest level of subjective wellbeing.

**TABLE 7:** Analysis of differences in subjective wellbeing by level of sports participation volume.

Variable	Group	N	M ± SD	*F*
Subjective wellbeing	Low exercise volume	587	2.89 ± 1.15	8.714***
	Moderate exercise volume	274	3.23 ± 0.95	
	High exercise volume	139	3.00 ± 1.05	

*p < 0.05, **p < 0.01,

***p < 0.001.

### 4.5 Structural equation model and research hypothesis testing

To further examine the relationships among sports participation, sports-related social support, basic psychological needs, and subjective wellbeing, as well as the pathways linking them, structural equation modeling was conducted using AMOS to test the hypothesized model.

#### 4.5.1 Path analysis of sports participation, sports-related social support, basic psychological needs, and subjective wellbeing

**FIGURE 2:**
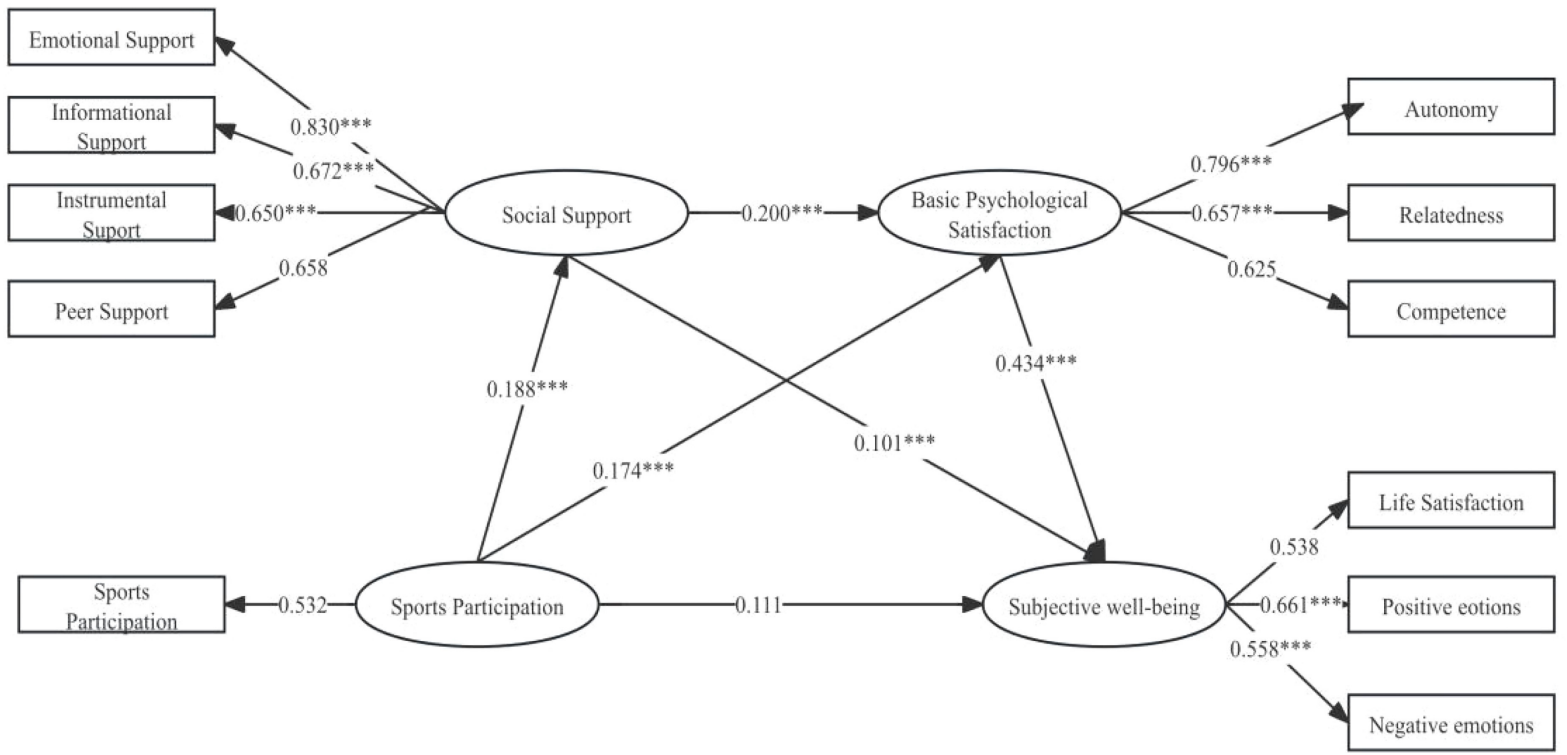
Structural equation model of sports participation, social support, basic psychological needs, and subjective wellbeing. *p < 0.05, **p < 0.01, ***p < 0.001.

To examine the relationships among sports participation, social support, basic psychological needs, and subjective wellbeing, a structural equation model was employed for analysis. The results are presented in Table 8 and Figure 2. The model fit indices indicated that all criteria met good standards (χ^2^/df = 1.649, CFI = 0.989, TLI = 0.985, RMSEA = 0.025), suggesting a good model fit. The path analysis results showed that sports participation significantly and positively predicted social support (β = 0.188, p = 0.001) and basic psychological needs (β = 0.174, *p* = 0.004). However, the direct path from sports participation to subjective wellbeing was not significant (β = 0.111, *p* = 0.129). Furthermore, social support not only significantly predicted basic psychological needs (β = 0.200, *p* < 0.001) but also had a significant positive effect on subjective wellbeing (β = 0.101, *p* = 0.027). Basic psychological needs had a significant effect on subjective wellbeing (β = 0.434, *p* < 0.001). In summary, sports participation was associated with subjective wellbeing through social support and basic psychological needs, respectively, and the chain mediation was supported, whereas the direct effect of sports participation on subjective wellbeing was not supported. Therefore, research hypothesis H1 was rejected, while H2 and H3 were supported.

**TABLE 8:** Fit indices of the structural equation model.

Common index	χ^2^	df	*p*	χ^2^/df	GFI	RMSEA	RMR	CFI	NFI	NNFI
Criterion	–	–	> 0.05	< 3	> 0.9	< 0.10	<0.05	> 0.9	>0.9	> 0.9
Value	62.677	38	0.007	1.649	0.988	0.025	0.024	0.989	0.974	0.985
Other Index	TLI	AGFI	IFI	PGFI	PNFI	PCFI	SRMR	RMSEA 90% CI		
Criterion	> 0.9	>0.9	> 0.9	>0.5	> 0.5	>0.5	< 0.1	–		
Value	0.985	0.980	0.989	0.569	0.673	0.684	0.024	0.013 ∼ 0.036		

#### 4.5.2 Mediation effect testing

With demographic variables controlled, the macro program Process was used to test the chain mediation model, with 5,000 bootstrap resamples for correction. The mediation effects of social support and basic psychological needs, as well as the chain mediation effect, were tested separately. As shown in Table 9, the total effect had an effect size of 0.224, with a confidence interval of [0.067, 0.381], indicating that the total effect was significant. The direct effect size was 0.112, accounting for 50% of the total effect, but its confidence interval was [-0.033, 0.257], which includes 0; therefore, the direct effect was not significant in this study. The first mediation pathway: Sports participation → Social support → Subjective wellbeing (Path 1) had an indirect effect size of 0.019, with a confidence interval of [0.001, 0.039], indicating that the indirect effect of Path 1 was significant. The second mediation pathway: Sports participation → Basic psychological needs → Subjective wellbeing (Path 2) had an indirect effect size of 0.076, with a confidence interval of [0.021, 0.132], indicating that the indirect effect of Path 2 was significant. The third mediation pathway: Sports participation → Social support → Basic psychological needs → Subjective wellbeing (Path 3) had an indirect effect size of 0.017, with a confidence interval of [0.004, 0.029], indicating that the indirect effect of Path 3 was significant. That is, sports participation is positively related to adolescents’ subjective wellbeing through the chain mediation of sports-related social support and basic psychological need satisfaction. Therefore, research hypothesis H4 was supported.

**TABLE 9:** Mediation Effects of social support and basic psychological needs.

Effect type	Path	Effect value	Standard error	95% CI lower limit	95% CI upper limit
Direct path	Sports participation → subjective wellbeing	0.112	0.074	–0.033	0.257
Indirect path 1	Sports participation → social support → subjective wellbeing	0.019	0.010	0.001	0.039
Indirect path 2	Sports participation → basic psychological needs → subjective wellbeing	0.076	0.028	0.021	0.132
Indirect path 3	Sports participation → social support → basic psychological needs → subjective wellbeing	0.017	0.006	0.004	0.029
Total indirect effect	Sum of all indirect path effects	0.112	0.031	0.052	0.173
Total effect	Sum of direct effect and total indirect effect	0.224	0.080	0.067	0.381

## 5 Discussion

### 5.1 Differences by gender, sport type, and only-child status

First, no significant gender differences were observed across the core study variables. This finding suggests that gender disparities in sports participation among adolescents may be gradually diminishing. Traditionally, males have often been granted greater opportunities and social acceptance for engaging in sports, whereas females have faced more restrictions due to prevailing gender norms and stereotypes. The present findings indicate that, with the advancement of modern educational concepts and the increasing emphasis on gender equality in sports, opportunities for female adolescents to participate in physical activities have been further expanded, and traditional gender stereotypes in the field of sports may be gradually weakening. Moreover, the positive psychological benefits associated with sports participation appear to be universal across genders, suggesting that both male and female adolescents can gain comparable physical and psychological benefits through engaging in sports. Second, regarding sport type, team sports may provide richer opportunities for peer interaction and stronger experiences of belonging, which may facilitate greater social support and the satisfaction of basic psychological needs. However, the group comparisons in the present study revealed no significant differences between individual and team sports across the core study variables. One possible explanation is that social support may not be solely determined by whether a sport is performed individually or collectively but may instead arise from the combined influence of multiple sources, including family members, peers, and coaches. Finally, regarding only-child status, only children scored significantly higher than non-only children in the dimension of social support. One possible explanation is that, as the sole child in the family, only children may receive more concentrated parental support during sports participation, such as parental accompaniment during exercise and greater provision of sports-related resources and equipment. In contrast, parental attention and family resources in non-only-child families may be distributed among multiple siblings. However, non-only children did not demonstrate lower levels of subjective wellbeing, suggesting the existence of a potential compensatory mechanism. Specifically, interactions with siblings may provide non-only children with natural opportunities for companionship and emotional exchange, and this horizontal support from siblings may compensate for relatively lower levels of parental support.

### 5.2 Association between sports participation on subjective wellbeing

The analysis revealed a statistically significant, albeit weak, association between sports participation and subjective wellbeing (*r* = 0.091), which is consistent with the findings of Wheatley and Bickerton (2017). However, the structural equation model showed that the direct effect of Sports Participation on subjective wellbeing was not statistically significant. Subjective wellbeing is a multifaceted construct that reflects individuals’ overall evaluations and experiences of their lives. It is influenced by multiple factors, including family environment, peer relationships, academic demands, personality characteristics, and socioeconomic conditions. As sports participation represents only one of many factors associated with subjective wellbeing, a relatively weak association between the two variables is not unexpected. This finding corrects the simplistic “exercise leads to happiness” logic found in previous studies and suggests the need for an in-depth analysis of how different exercise volumes affect adolescents’ subjective wellbeing. Analyzing the effect of sports participation on subjective wellbeing in this study, moderate exercise volume was found to have the optimal effect on promoting subjective wellbeing, whereas subjective wellbeing decreased when exercise volume was reduced or increased. This result is consistent with the findings of Huizhen (2009). Specifically, for adolescents with “low exercise volume,” their sports participation is often characterized by low frequency and low intensity of physical activity. Exercise-induced mood improvement is highly dependent on a specific level of physiological arousal. For example, the secretion of BDNF and endorphins typically requires achieving moderate-to-high intensity aerobic load sustained over a certain period to effectively activate the reward circuitry of the limbic system (Mielniczek and Aune, 2024). Low-intensity sports participation struggles to surpass this physiological threshold and fails to produce a noticeable sense of pleasure. Therefore, low-volume sports participation cannot directly drive improvements in subjective wellbeing. Regarding “high exercise volume,” although moderate exercise benefits health, excessive and frequent sports participation may lead to negative effects. High volumes of exercise may lead to the accumulation of physical fatigue; if recovery is delayed, this may contribute to elevated levels of stress hormones, such as cortisol, thereby triggering physiological stress responses. Consequently, high-intensity sports participation may become a source of stress, offsetting the potential psychological benefits of exercise itself. In addition, previous studies have predominantly employed regression analysis and meta-analysis, focusing primarily on the overall association between sports participation and subjective wellbeing, while paying relatively little attention to the inclusion of other mediating variables. However, once mediating variables are incorporated, the effect of sports participation on subjective wellbeing may change substantially.

Self-determination theory may provide a useful framework for understanding why the direct association between sports participation and subjective wellbeing was not significant and why the observed relationship between the two variables was relatively weak.

One possible explanation for this is that a significant portion of physical activity among adolescents in China currently stems from mandatory physical education classes in schools. When behavior is driven by “having to do it” rather than “wanting to do it,” even if the exercise volume reaches moderate levels, participation under such controlled motivation is unlikely to generate subjective wellbeing. This further explains why basic psychological needs must be introduced as mediators. Only when the process of sports participation satisfies adolescents’ need for autonomy—transforming “being forced to exercise” into “wanting to exercise”—can sports participation be converted into subjective wellbeing. However, these explanations were not empirically tested in the present study, and the influence of compulsory school-based physical education remains speculative. Future research is therefore needed to directly examine these potential mechanisms.

### 5.3 The mediating role of social support between sports participation and subjective wellbeing

The study found that the indirect association between sports participation and subjective wellbeing through social support was significant, suggesting that social support may play an important role in the association between physical activity and subjective wellbeing. First, sports activities are a crucial source of social support for adolescents. Sports activities have clear role divisions and team goals, requiring participants to communicate and cooperate. Whether in team sports such as basketball and soccer or individual sports such as running and fitness, sports participation offers adolescents a connection based on shared interests. This connection has significantly expanded the breadth and depth of adolescents’ social networks (Bang et al., 2024). Moreover, interactions within sports contexts are characterized by strong emotional involvement. Sharing the joy of victory, the frustration of defeat, and the challenges of training easily fosters emotional resonance among peers. Relationships built on such shared experiences tend to be more resilient than ordinary interpersonal relationships, enabling adolescents to perceive higher levels of peer support and emotional support. This explains the strong correlations found in this study between sports participation and the dimensions of “companion support” and “emotional support.” Second, social support significantly and positively predicts subjective wellbeing. This further supports the core value of social support as an individual psychological resource. From the perspective of the main effect model, social support itself is a positive psychological experience. According to Maslow’s hierarchy of needs (Maslow and Lewis, 1987), the need for love and belongingness is a fundamental human motivation. When adolescents perceive affirmation from coaches (evaluative support), encouragement from teammates (emotional support), and assistance in times of difficulty (instrumental support) through sports participation, this perception of “being accepted and cared for” directly enhances their life satisfaction and positive affect. From the perspective of the buffering effect model (Lin et al., 1985), adolescence is a sensitive period characterized by physical and emotional turbulence coupled with academic pressure. When adolescents encounter setbacks, strong social support provides resources for cognitive reappraisal, reducing the negative impact of stressful events on adolescents and thereby maintaining higher levels of subjective wellbeing.

### 5.4 The mediating role of basic psychological needs between sports participation and subjective wellbeing

The study shows that the association between sports participation and subjective wellbeing is mediated by basic psychological need satisfaction. First, sports participation is the most direct domain for individuals to acquire a sense of competence. Compared with the delayed feedback of academic performance (e.g., final exams), sports activities offer highly immediate feedback. Every successful shot or instance of tactical cooperation provides adolescents with positive feedback. This high-frequency experience of success directly strengthens their sense of competence. Furthermore, sports participation includes not only success but also failure. In competitive sports contexts, while adolescents gain a sense of competence, they also learn how to face “competence frustration.” Attribution theory (Kelley and Michela, 1980) suggests that making appropriate attributions for failure in sports activities helps adolescents attribute failure to controllable factors such as “insufficient effort” rather than uncontrollable factors such as “lack of ability.” This psychological process of continuously affirming one’s abilities through ongoing challenges greatly satisfies the need for competence and allows this confidence to transfer to academic and life domains, thereby enhancing subjective wellbeing. Second, satisfaction of the need for autonomy is a key pathway to improving subjective wellbeing. SDT posits that wellbeing depends on the nature of behavioral motivation. Although some school physical education is compulsory, in the broader context of sports participation—particularly extracurricular sports activities—adolescents have more autonomy in making choices. Such interest-based choices are an important manifestation of autonomous decision-making in individual behavior. When adolescents experience their sports participation as “voluntary” rather than “externally coerced,” their perceived locus of causality shifts from external to internal. This process of self-integration allows individuals to experience genuine psychological freedom, which directly nurtures the need for autonomy, making sports participation an important avenue for adolescents to relieve stress and find themselves, thereby translating into high levels of subjective wellbeing. Finally, sports participation creates a unique empathic field. In the process of team cooperation, adolescents not only receive help from teammates but also experience being “needed and accepted.”

### 5.5 Chain mediating role

The study indicated that social support and basic psychological need satisfaction sequentially mediated the association between sports participation and adolescents’ subjective wellbeing. It should be noted that, although the serial mediation pathway reached statistical significance, its effect size was relatively small. This finding suggests that, while social support and basic psychological needs constitute important factors underlying the association between sports participation and adolescents’ subjective wellbeing, they are not the only pathways involved. First, guidance from coaches, collaboration with teammates, and family support for sports participation can constitute a high-quality social support network, and this external interpersonal interaction serves as the physiological and psychological foundation for satisfying the need for “relatedness.” According to social belonging theory (Baumeister and Leary, 1995), when adolescents perceive being cared for and accepted in sports participation, this external “support resource” is directly internalized as a psychological sense of “connectedness,” thereby achieving the immediate transformation of need satisfaction. At the same time, positive social support can significantly reduce adolescents’ fear of failure and enhance their self-efficacy in the process of skill acquisition. Second, after external social support is successfully transformed into satisfaction of basic psychological needs through individuals’ cognitive processing, the generation of wellbeing enters the “internal drive stage.” This study found that basic psychological needs had a relatively strong direct effect on subjective wellbeing within the chain model. According to SDT, basic psychological needs are “essential nutrients” for individual happiness. Although social support provides abundant external resources, if these resources cannot be transformed into individuals’ confirmation of their own abilities (competence), experience of interpersonal interaction (relatedness), and control over behavioral volition (autonomy), then the effect of social support will be greatly diminished. The chain mediating pathway implies that the social interaction brought about by sports participation ultimately returns to the individual’s self-system, achieving positive emotional transformation through the satisfaction of psychological needs. This may further explains why the direct effect of sports participation was not significant in this study: primarily because exercise that does not undergo the integration of social resources and the internalization of psychological needs cannot reach the core of individual wellbeing. Finally, the chain mediation model is examined from a holistic perspective. For a long time, sports have been misunderstood as merely physical labor. This study, through the lens of chain mediation, demonstrates that sports are also about “mental training” This “mental training” does not happen overnight but occurs through a delicate transmission from “capitalization of social relationships” to “internalization of psychological needs.” This finding highlights the importance of “environmental cultivation” in physical education. Against the backdrop of intense academic competition, sports participation not only provides opportunities for exercise but, more importantly, establishes a buffer zone through physical activity. Within this buffer zone, adolescents resist external pressure by obtaining social support and rediscover themselves in the process of need satisfaction.

## 6 Conclusion and recommendations

### 6.1 Conclusion

(1) The direct effect of sports participation on subjective wellbeing is not significant. This suggests that sports participation does not promote subjective wellbeing simply through the accumulation of exercise volume. (2) Social support plays a mediating role between sports participation and subjective wellbeing. Sports participation can significantly enhance adolescents’ perception of social support and further promote subjective wellbeing. (3) Satisfaction of basic psychological needs plays a mediating role between sports participation and subjective wellbeing. Sports participation significantly predicts satisfaction of basic psychological needs and further enhances subjective wellbeing through need satisfaction. This finding aligns with the core tenet of SDT: the enhancement of wellbeing is not a passive outcome of external events but a stable psychological benefit derived from the continuous acquisition of autonomy, competence, and relatedness during activities. (4) The most explanatory finding of this study is the chain mediating pathway, whereby sports participation first enhances social support, which further promotes satisfaction of basic psychological needs, and ultimately improves subjective wellbeing. (5)It should be noted that the serial mediation pathway identified in this study represents a pattern of statistical associations and should not be interpreted as evidence of causality. Given the cross-sectional nature of the data, the temporal ordering of the variables cannot be established.

### 6.2 Recommendations

#### 6.2.1 School level: constructing a “need-supportive” physical education ecosystem

Given that social support emerged as a significant mediator in our model, it seems reasonable to consider the social aspects of physical education more carefully. For instance, structuring class activities around cooperative tasks or team competitions—where students must rely on one another—might help build a stronger sense of community among peers. Beyond the social layer, our finding that basic psychological needs satisfaction also mediated the participation–wellbeing link points to another practical consideration: when adolescents feel a sense of choice in what they do physically, their engagement may become more personally meaningful. Letting students pick from several activity options rather than prescribing a single uniform exercise could be one modest way to support this.

#### 6.2.2 Community level: improving the compensation mechanism for social capital

First, it is recommended to establish youth sports clubs in community public spaces, focusing on providing low-threshold opportunities for sports participation, especially for children from multi-child families. Second, a “family-school-community” collaborative sports culture field should be constructed. Through parent-child sports events, community leagues, and other formats, sports participation should be embedded within broad social relationship networks. This comprehensive support can increase adolescents’ social embeddedness in sports, enabling them to perceive support from significant others across different settings and maximize the cumulative effect of social capital.

#### 6.2.3 Individual level: enhancing the “reflectiveness” and “proactiveness” of sports participation

Adolescents should elevate their cognitive understanding of the value of sports, shifting from simple physical exercise to psychological resource management. During participation, they should consciously use the sports context to develop interpersonal relationships, actively seek team cooperation, and engage in peer assistance. At the same time, they should learn to make positive attributions in athletic challenges and transfer the sense of mastery gained from sports to daily life, thereby cultivating positive psychological qualities.

## 7 Research limitations and future directions

(1) Limitations in causal inference and the need for longitudinal research. This study is based on cross-sectional data; although the SEM model validated the direction of the pathways, it cannot completely rule out the possibility of reverse causality. Future research should employ cross-lagged designs or longitudinal studies, collecting data at multiple time points to accurately capture the dynamic evolutionary patterns and causal directions among variables.

(2) Given that this study adopts a cross-sectional design and the data were collected from self-reported measures at a single time point, the conclusions and implications drawn in this study should be interpreted with caution when being generalized to other contexts. This also constitutes one of the limitations of the present study.

(3) This study analyzed sports participation only in terms of team and individual sports, without distinguishing between competitive and recreational activities, nor did it examine whether participation was voluntary. These distinctions may elicit different psychological responses among participants; future research could explore these issues by incorporating motivational variables or employing other research tools (such as questionnaires).

(4) This study employed non-probability convenience sampling. Although the sample was drawn from multiple provinces and various types of schools, it may still not fully represent the general population of Chinese adolescents. Therefore, caution should be exercised when generalizing the findings to adolescents in different regions and socioeconomic contexts. Future studies may adopt probability sampling methods, such as stratified sampling or multistage cluster sampling, to enhance the representativeness of the sample and the external validity of the research conclusions.

(5) In this study, all variables were measured using self-report questionnaires administered at a single point in time. However, since social support, basic psychological needs, and subjective wellbeing are inherently subjective psychological perceptions, self-report measures remain the most appropriate method; consequently, it is practically difficult to completely eliminate common-method bias in such studies. Future research could further mitigate this bias through longitudinal designs and the collection of data from multiple sources.

(6) This study adopted the WHO definition of adolescence (10–19 years), covering multiple educational stages. However, differences across educational stages were not examined through separate SEM analyses, which warrants further investigation in future studies.

## Data Availability

The raw data supporting the conclusions of this article will be made available by the authors, without undue reservation.
